# Patterns of host-parasite associations between marine meiofaunal flatworms (Platyhelminthes) and rhytidocystids (Apicomplexa)

**DOI:** 10.1038/s41598-023-48233-y

**Published:** 2023-11-29

**Authors:** Niels W. L. Van Steenkiste, Kevin C. Wakeman, Bill Söderström, Brian S. Leander

**Affiliations:** 1 Departments of Botany and Zoology, University of British Columbia, Vancouver, BC Canada; 2 Hakai Institute, Heriot Bay, Quadra Island, BC Canada; 3 Institute for the Advancement of High Education, Hokkaido University, Sapporo, Japan; 4 Australian Institute for Microbiology and Infection, University of Technology Sydney, Ultimo, Australia

**Keywords:** Evolution, Zoology

## Abstract

Microturbellarians are abundant and ubiquitous members of marine meiofaunal communities around the world. Because of their small body size, these microscopic animals are rarely considered as hosts for parasitic organisms. Indeed, many protists, both free-living and parasitic ones, equal or surpass meiofaunal animals in size. Despite several anecdotal records of “gregarines”, “sporozoans”, and “apicomplexans” parasitizing microturbellarians in the literature—some of them dating back to the nineteenth century—these single-celled parasites have never been identified and characterized. More recently, the sequencing of eukaryotic microbiomes in microscopic invertebrates have revealed a hidden diversity of protist parasites infecting microturbellarians and other meiofaunal animals. Here we show that apicomplexans isolated from twelve taxonomically diverse rhabdocoel taxa and one species of proseriate collected in four geographically distinct areas around the Pacific Ocean (Okinawa, Hokkaido, and British Columbia) and the Caribbean Sea (Curaçao) all belong to the apicomplexan genus *Rhytidocystis*. Based on comprehensive molecular phylogenies of Rhabdocoela and Proseriata inferred from both 18S and 28S rDNA sequences, as well as a molecular phylogeny of Marosporida inferred from 18S rDNA sequences, we determine the phylogenetic positions of the microturbellarian hosts and their parasites. Multiple lines of evidence, including morphological and molecular data, show that at least nine new species of *Rhytidocystis* infect the microturbellarian hosts collected in this study, more than doubling the number of previously recognized species of *Rhytidocystis*, all of which infect polychaete hosts. A cophylogenetic analysis examining patterns of phylosymbiosis between hosts and parasites suggests a complex picture of overall incongruence between host and parasite phylogenies, and varying degrees of geographic signals and taxon specificity.

## Introduction

Apicomplexans are one of the most diverse and ubiquitous groups of unicellular eukaryotes infecting animal hosts. While some apicomplexans are well-known pathogens of humans and domesticated animals (e.g., *Toxoplasma*, *Cryptosporidium* and *Plasmodium*), a large and mostly undescribed diversity of these parasites are present in marine animals^1^. For instance, gregarines are widespread but poorly studied parasites of larger marine invertebrates including annelids, molluscs, nemerteans, phoronids, hemichordates, tunicates and arthropods^2^. However, research on apicomplexan parasites of microscopic marine invertebrates, such as zooplankton and meiofauna, is very limited.

Meiofauna constitute an important reservoir of marine biodiversity, comprising taxonomically diverse and extremely abundant communities of microscopic invertebrates associated with sediments and other substrates (e.g., macroalgae) in almost every marine habitat around the world^3^. Many of these microscopic animals are in the same size range of unicellular eukaryotes and therefore hardly considered as hosts for parasitic protists. Indeed, comprehensive studies on parasites of meiofauna are almost completely lacking^4^. One recent study looking into symbiont eukaryotic microbiomes of microscopic marine invertebrates showed an unexpected diversity of apicomplexans in a variety of host taxa^5^. Given the important roles of meiofauna in marine ecosystems^6^ and the impact that parasites could have on various elements of their hosts’ life history (e.g., growth and reproduction)^7^, studies on both meiofauna diversity and their parasites can provide us with additional insight into the ecology and evolution of interstitial communities in particular and marine ecosystems as a whole.

Most animal phyla contain microscopic representatives, and several phyla are exclusively meiofaunal (e.g., Loricifera, Kinorhyncha, Gnathostomulida, and Gastrotricha)^8^. Meiofaunal flatworms or so-called "microturbellarians" are among the most abundant and diverse groups in these meiofaunal communities^9,10^, with the vast majority of microturbellarian species remaining to be discovered^11,12^. As predators of microalgae, other meiofauna and smaller macrofauna, microturbellarians likely play an important role in structuring the communities of various marine habitats^13^. However, our knowledge on ecological interactions between microturbellarians and other organisms, including their symbionts and parasites, remains highly anecdotal.

A variety of protists, including apicomplexans, are known to infect flatworms. More general overviews on turbellarians and their parasites list larger-sized freshwater triclads and marine polyclads as hosts, with very few references to microturbellarians^14,15^. Nevertheless, several taxonomic works on marine or brackish water microturbellarians over the past 140 years have mentioned apicomplexans parasitizing these tiny animals, and either define these infectious cells as “gregarines”^16–23^ (German: Gregarinen), “sporozoans”^24,25^ (German: Sporozoen) or “apicomplexans”^26^. Attempts to further identify and characterize these parasites of marine microturbellarians have never been undertaken except for one study by Cannon and Jennings (1988), in which the hyperparasitic gregarine *Monocystella epibatis* Cannon & Jennings, 1988 of a pterastericolid rhabdocoel that is endobiotic in the crown-of-thorns starfish was described^27^.

In this study, we explore the diversity, identity and host specificity of apicomplexans infecting rhabdocoels and proseriate microturbellarians collected from three geographically distinct locations around the Pacific Ocean and from one location in the Caribbean. In addition to morphological data, we provide comprehensive molecular phylogenies of the hosts inferred from both 18S and 28S rDNA sequences, as well as a molecular phylogeny of the parasites inferred from 18S rDNA sequences. A cophylogenetic analysis examined potential patterns of phylosymbiosis between hosts and parasites.

## Material and methods

**Figure 1:**
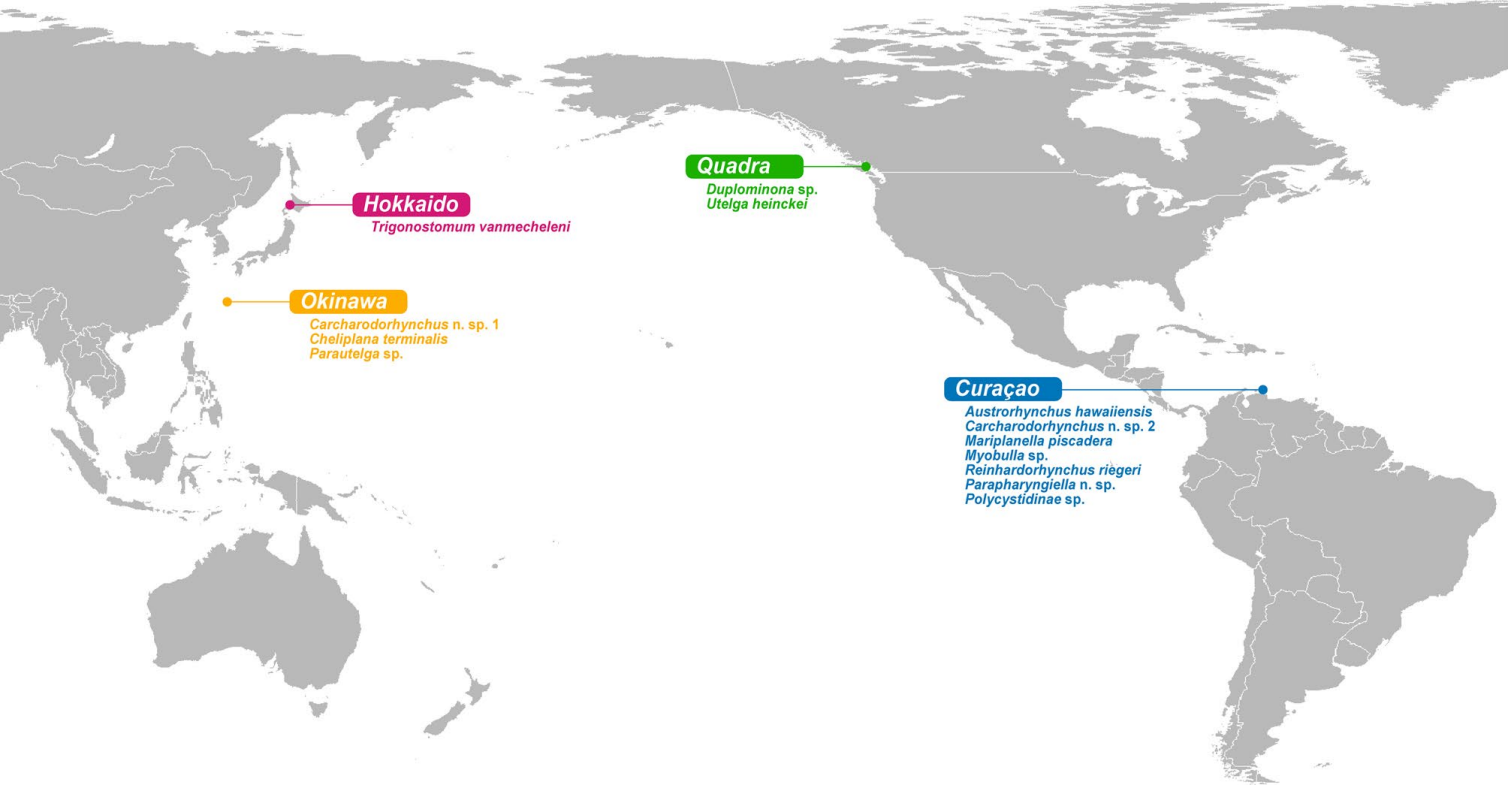
Sampling locations of microturbellarian hosts in this study. For each location, the flatworm species found to be infected with rhytidocystids are listed. The original map can be found at https://en.m.wikipedia.org/wiki/File:Pacific-centric-map.png and was modified in Adobe Illustrator v26.2.1.

**Figure 2:**
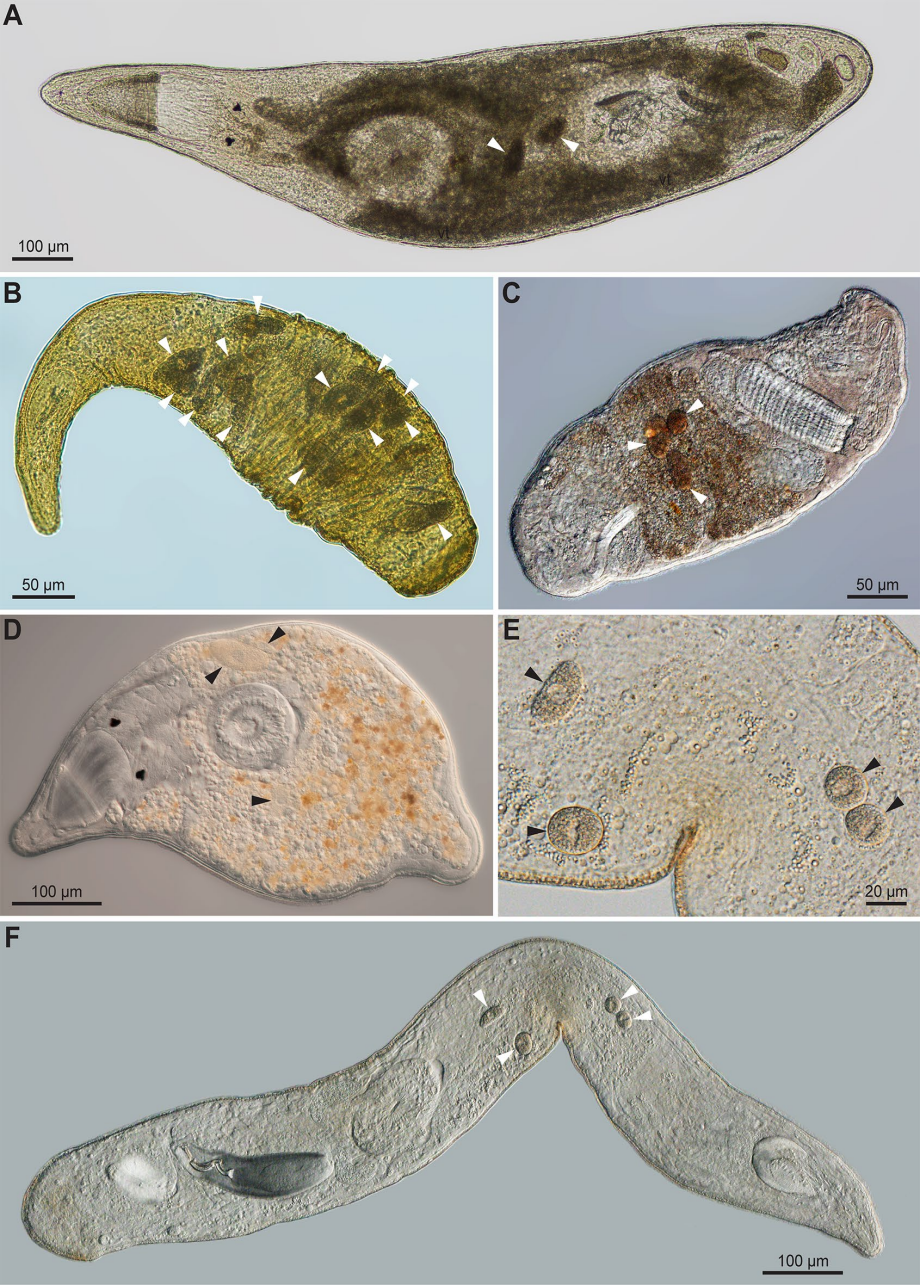
Light micrographs of microturbellarians infected with rhytidocystids. (**A**) *Reinhardorhynchus riegeri* from Curaçao: host specimen and parasites used for DNA sequencing (CU1272 in Tables S3–S4). (**B**) *Carcharodorhynchus* n. sp. 2 from Curaçao: host specimen and parasites used for DNA sequencing (CU271 in Tables S3–S4). (**C**) *Cheliplana terminalis* from Okinawa: host tissue used for DNA sequencing (OK273 in Table S3), isolated parasite cells used for SEM (Fig. 3A) and DNA sequencing (OK1 in Table S4). (**D**) *Utelga heinckei* from Quadra: host specimen and parasites used for DNA sequencing (QU4 in Tables S3–S4). (**E**,**F**) *Carcharodorhynhcus* n. sp. 1 from Okinawa: one of two host specimens belonging to the same species, the other one used for DNA sequencing (OK293 in Table S3) and parasite isolation for SEM (Fig. 3C). Cells of *Rhytidocystis* are indicated by black or white arrowheads.

Meiofaunal flatworm hosts were collected during a series of sampling campaigns to the islands of Hokkaido and Okinawa (Japan), Curaçao (Kingdom of the Netherlands), and Quadra Island (British Columbia, Canada) from 2016 to 2022 (Fig. 1). Collection data are provided in Table S1. The substrates, either sand or algae, were sampled by hand in the intertidal or the subtidal while snorkeling. Microturbellarians were separated from the sediment using the MgCl_2_ decantation method^28^. Individual worms were isolated and observed under a stereoscope and subsequently whole mounted alive to be visually inspected for the presence of parasites using a compound microscope. Infected individuals were photographed (Fig. 2A–F) and retained for further analysis. The flatworm hosts were identified based on morphological characters visible in the live animals. For five out of seventeen host specimens, i.e., *Duplominona* sp., *Parautelga* sp., *Polycystidinae* sp., and the two specimens of *Myobulla* sp., too few characters could be observed to identify them to species level. Another four specimens, i.e., *Carcharodorhynchus* n. sp. 1, *Carcharodorhynchus* n. sp. 2, and the two specimens of *Parapharyngiella* n. sp., most likely belong to three new species, since the diagnostic characters could not be matched with any of their known congeners.

**Figure 3:**
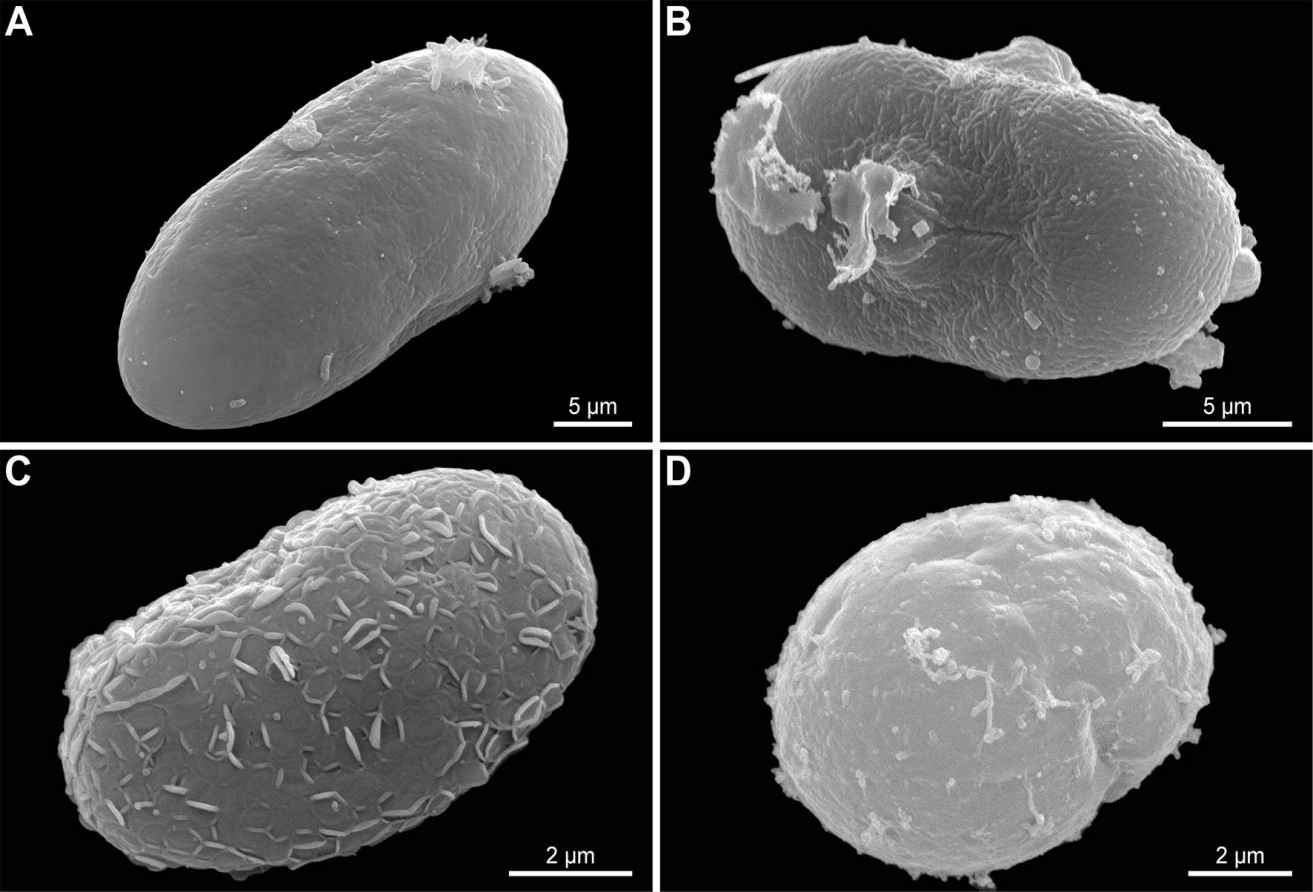
Scanning electron micrographs of rhytidocystid cells from microturbellarian hosts. (**A**) Species a from *Cheliplana terminalis*. (**B**) Species h from *Parautelga* sp. (**C**) Species f from *Carcharodorhynchus* n. sp. 1. (**D**) Species c from *Trigonostomum vanmecheleni*.

Some apicomplexan trophozoites isolated from microturbellarian hosts from Japan were prepared for scanning electron microscopy (Fig. 3A–D). Rhabdocoels were broken apart by micro-dissection under an inverted microscope (Olympus CKX-53) (Tokyo, Japan) using a pair of glass pipettes that were hand-drawn to a fine point. The isolated parasite cells, now freed from the tissue, were washed two times (until clean), and then placed on 3 µm Merk Millipore membrane filters (Massachusetts, USA) and fixed with 2.5% glutaraldehyde in seawater. Subsequent dehydration, critical point drying, and imaging was done following Wakeman (2020)^29^.

Genomic DNA was extracted from entire host worms and host tissue using the DNeasy Blood & Tissue kit (Qiagen). Manufacturer’s instructions were followed, with the exception that DNA was eluted in 60 μl of preheated AE elution buffer (60 °C). Fragments of the nearly complete 18S (amplicon size range: 1747–2043 bp) and partial 28S rRNA (amplicon size range: 1660–1821 bp) genes of the host and of the 18S rRNA gene of the parasite (amplicon size range: 1154–1708 bp) were PCR amplified using the primers and thermocycling conditions in Table S2. Amplicons were visualized on 1.5% agarose gels stained with GelRed™ (Biotium), enzymatically cleaned with Illustra™ ExoProStar S (GE Healthcare), and subsequently sequenced by Genewiz (Brooks Life Sciences) through standard Sanger DNA sequencing using the amplification primers and internal sequencing primers in Table S2. From the flatworms collected in Hokkaido and Okinawa, Japan, single *Rhytidocystis* cells were isolated (and washed in sterilized seawater) using hand-drawn glass pipettes on a CKX-53 inverted microscope (Olympus). Genomic DNA was prepared from these samples using 20 μl FFPE (Lucigen) solution, following the manufacturer’s instructions. The 18S rRNA gene from these single-cell isolates were amplified in a nested reaction with KOD One^®^ PCR Master Mix (Toyobo). The resulting amplicons were visualized on 1.5% agarose gels stained with ethidium bromide, PEG-purified, and sequenced following Wakeman (2020)^29^. Nested PCR conditions and primers are detailed in Table S2. Resulting trace files were assembled into full sequences in Geneious v11.0.15^30^ and subjected to a BLAST search on the NCBI website (http://blast.ncbi.nlm.nih.gov) to verify the taxonomic identity of the hosts and parasites. Sequences were deposited in GenBank under accession numbers detailed in Tables S3 and S4.

The new 18S and 28S rDNA sequences of the rhabdocoel hosts were aligned with rDNA sequences of 110 and 101 representatives of other rhabdocoels, respectively, and with rDNA sequences of three proseriates as an outgroup (Table S3). The 18S and 28S rDNA sequences of the proseriate host *Duplimona* sp. were aligned with rDNA sequences of 25 representatives of other proseriates, and of three rhabdocoels as an outgroup (Table S3). The 17 new 18S rDNA sequences belonging to representatives of the apicomplexan genus *Rhytidocystis* (Table S4) were aligned with 53 18S rDNA sequences of 52 members of other apicomplexan, squirmid and chrompodellid taxa (Table S5). The 28S rDNA sequences of 28 apicomplexan, squirmid and chrompodellid representatives were also used in the phylogenetic analyses. The outgroup taxa were selected based on current knowledge of the phylogenetic relationships within flatworms^31,32^ and within apicomplexans^33,34^. All alignments were produced with the E-INSI algorithm in MAFFT^35^ and subsequently trimmed with ClipKIT using the default settings^36^. The trimmed 18S rDNA and 28S rDNA alignments were concatenated in Geneious v11.0.15 to produce two datasets for the flatworm hosts (rhabdocoels and proseriates) and one dataset for the parasites. Genetic similarities among and between putative species of *Rhytidocystis* were calculated in Geneious v11.0.15 based on a separate 1638 bp 18S rDNA alignment (MAFFT, E-INSI) containing all 25 *Rhytidocystis* sequences.

The best-fit partitions and models of molecular evolution corresponding to the 18S and 28S rDNA datasets and GTR + GAMMA + I were recovered for the concatenated (18S + 28S) datasets in PartitionFinder v.2.1.1 using a greedy search and all three model selection criteria (AIC, AICc, and BIC)^37^. This partition scheme and models were subsequently used in the phylogenetic analyses of the concatenated datasets. The maximum likelihood (ML) analyses were performed with the RAxML v8.2.11 plugin^38^ in Geneious v11.0.15 selecting the algorithm for best-scoring ML tree search and non-parametric bootstrapping (1000 replicates). Bayesian analyses were performed on the same datasets in MrBayes v3.2.7a^39^ through XSEDE in the CIPRES Science Gateway v3.3 (https://www.phylo.org), using default prior and MCMC settings, in two independent simultaneous runs for 40 million generations. Trees were sampled every 400^th^ generation after a 25% burnin. Convergence was assessed through the LogL values, ESS values (estimated sample size) and the average deviation of split frequencies. The remaining 75,000 trees were summarized in a 50% majority-rule consensus tree. Branch support was evaluated with the ML bootstrap values (bs) and Bayesian posterior probabilities (pp) for the ML and Bayesian trees, respectively.

Congruence between host and parasite phylogenies was investigated using the Procrustean Approach to Cophylogeny (PACo) method to compute a goodness-of-fit statistic based on the residual sum of squares^40^. The number of random permutations of the host-parasite association matrix was set to 100,000. In addition, PACo also assessed the contribution of each individual host-parasite association to the overall fit. To visualize host-parasite relationships, a tanglegram was manually drawn in Adobe Illustrator v26.2.1 (Adobe Inc.) based on the output of a tanglegram generated in Random Tanglegram Partitions (Random TaPas)^41^. The host and parasite input trees for PACo and Random TaPas were produced in MrBayes v3.2.7a in the same way as described above, but the alignments only included the seventeen 18S and 28S rDNA sequences of the flatworm hosts and seventeen 18S rDNA sequences of their apicomplexan parasites listed in Tables S1 and S4. Both PACo and Random TaPas scripts were run in Rstudio v2022.07.2.

### Sampling

Collection permits for the field work were obtained by the Carmabi Marine Research Institute (Curaçao) and through Fisheries and Oceans Canada (XR 224 2021).

## Results

### Apicomplexans are widespread parasites of meiofaunal flatworms

Our collections span a large geographical area in various tropical, subtropical and temperate marine ecoregions^42^: the Sea of Japan and the Georgia Basin in the Temperate Northern Pacific, the South Kuroshio region in the Central Indo-Pacific, and the southern Caribbean in the Tropical Atlantic (Fig. 1). Parasite prevalence could not be quantified, because flatworm hosts were sampled qualitatively. However, at least for one location, Quadra Island, qualitative collections have been performed on a regular basis since 2021. Both host taxa from this area, *Utelga heinckei* (Fig. 2D) and *Duplominona* sp. are regularly encountered in subtidal samples, especially during cooler months of the year. Despite the relative abundance of host species, apicomplexan parasites were only observed in three individuals of *U. heinckei* and one individual of *Duplominona* sp., suggesting the prevalence of recognizable apicomplexan life stages (e.g., larger trophozoites) is low (< 5%). A similarly low prevalence was observed in animals from Okinawa and Curaçao. Based on their appearance and location inside the worm host (gut), all the observed parasite cells are thought to be trophozoite stages.

### Molecular phylogenies of the hosts and parasites

**Figure 4:**
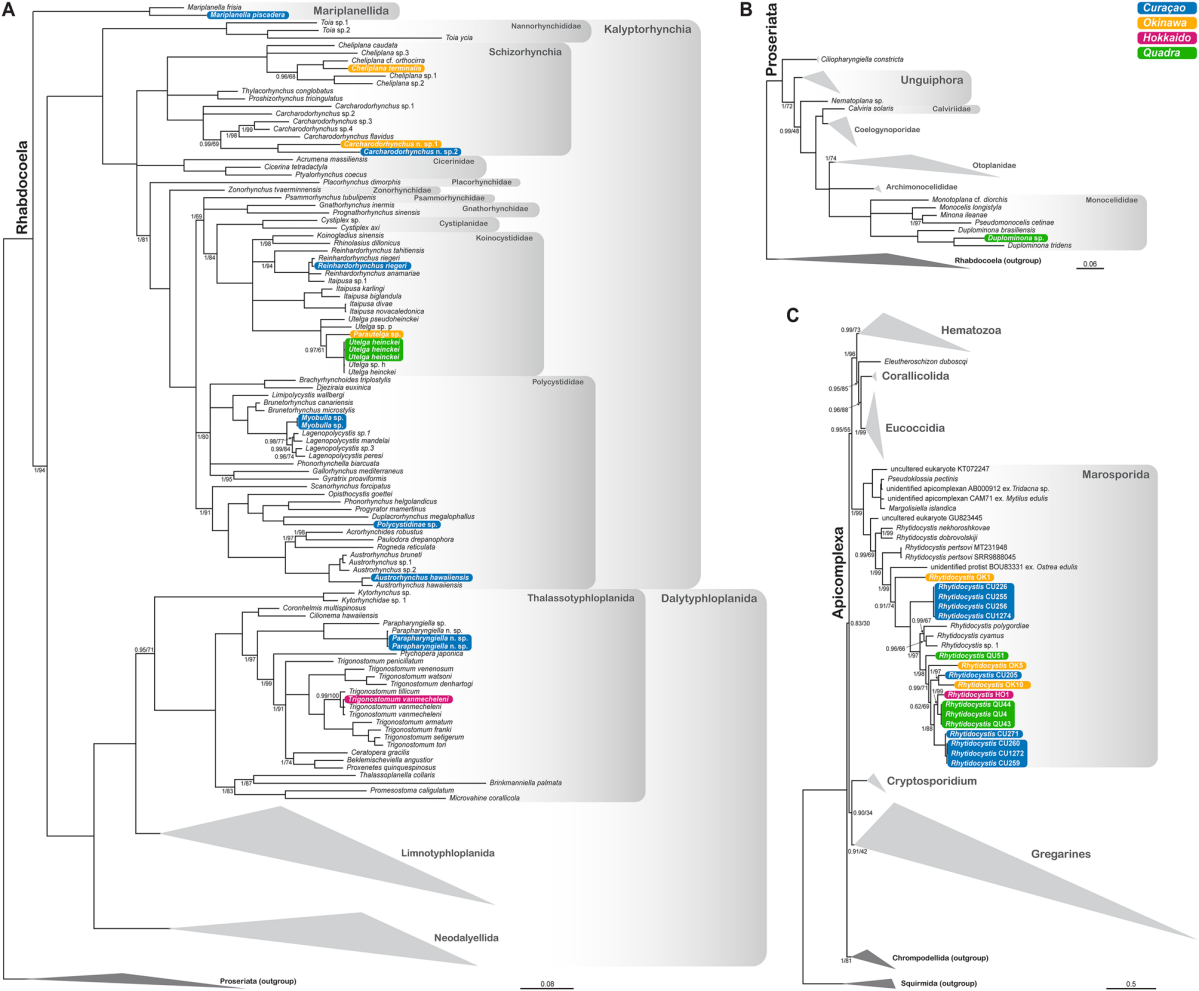
Phylogenetic positions of the microturbellarian hosts and their rhytidocystid parasites. Bayesian majority-rule consensus trees of the MrBayes analyses based on the trimmed concatenated alignment for (**A**) Rhabdocoela (Platyhelminthes), (**B**) Proseriata (Platyhelminthes), and (**C**) Apicomplexa. Branch support values of the Bayesian analyses (pp, posterior probabilities) and the topologically congruent ML analysis (bs, bootstrap) are not shown in the host trees when pp = 1 and bs = 100. Branches with support values of pp < 0.95 or bs < 70 are collapsed. All other branches with annotations have support values of pp ≥ 0.95 and/or bs ≥ 70.

The trimmed concatenated 18S + 28S rDNA sequence alignment for the rhabdocoel host phylogeny had 130 sequences belonging to 120 taxa and contained 3754 bp (18S: 1921 bp; 28S: 1833 bp), while the trimmed concatenated 18S + 28S rDNA sequence alignment for the proseriate host phylogeny had 29 sequences belonging to 28 taxa and contained 3484 bp (18S: 1776 bp; 28S: 1708 bp). The trimmed concatenated 18S + 28S rDNA sequence alignment for the apicomplexan parasite phylogeny had 70 sequences belonging to 61 taxa and contained 5858 bp (18S: 2076 bp; 28S: 3782 bp). The phylogenetic analyses to determine the positions of the flatworm hosts and their parasites among other rhabdocoel and proseriate flatworms, and among other apicomplexans, respectively, are illustrated in Fig. 4. Topologies were congruent for both the ML and Bayesian trees when branches with low support values (pp < 0.95 and bs < 70) were not taken into account.

The phylogenetic positions of the rhabdocoel and proseriate hosts largely correspond with our identifications based on morphological characters (Fig. 4A–B). Nearly all host taxa fell within clades corresponding to their respective genera, except for *Parautelga* sp., *Myobulla* sp., and *Polycystidinae* sp. Among rhabdocoels, the host taxa represent a wide taxonomic diversity, with representatives of nearly all major lineages, except for the predominantly freshwater limnotyphloplanids and the mostly marine neodalyellids (Fig. 4A).

**Figure 5:**
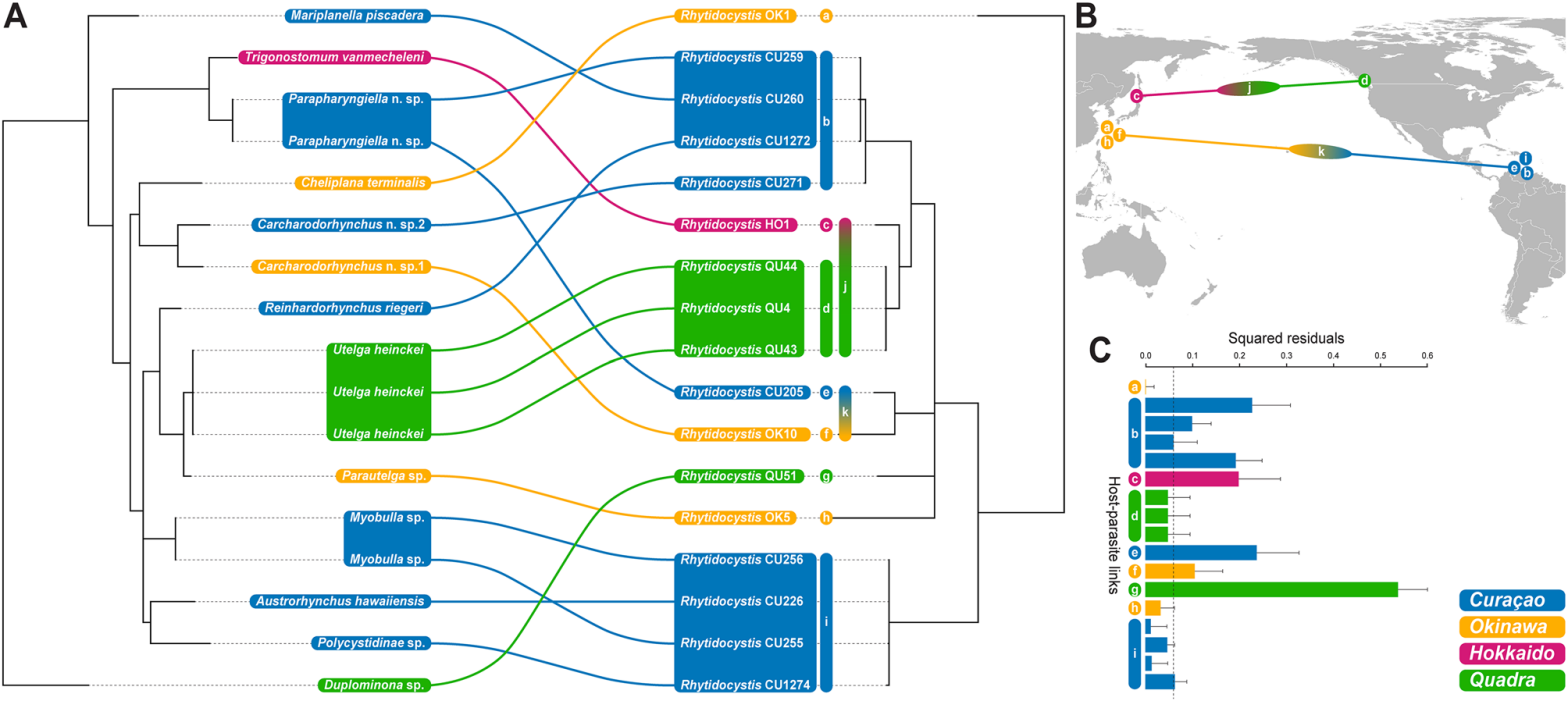
Host-parasite associations between microturbellarians and rhytidocystids. (**A**) Tanglegram showing an overall incongruence between host and parasite phylogenies. Species of *Rhytidocystis* are denoted a–i. (**B**) Sister group connections within clades j and k visualized on a map of the Pacific Ocean. (**C**) Contributions of each individual squared residual representing a host-parasite association to the overall goodness-of-fit statistic. The dashed line indicates the median squared residual value.

The phylogenetic analyses on the apicomplexan sequences clearly show that they belong to the apicomplexan genus *Rhytidocystis* within the group Marosporida (Fig. 4C)*.* The taxa infecting flatworms do not form a monophyletic assemblage, given the positions of *Rhytidocystis* sp. 1, *R. cyamus*, and *R. polygordiae* (all of which infect polychaete annelids). Four other representatives of *Rhytidocystis* in the tree, *R. dobrovolskiji*, *R. nekhoroshkovae*, and *Rhytidocystis pertsovi* are also intestinal parasites of polychaetes and form the more basal lineages within the genus. Among the flatworm parasites, several clusters of nearly identical haplotypes can be recognized based on genetic similarities: clade b (99.05–99.87%) and clade i (99.55–99.97%) from Curaçao, and clade d (100%) from Quadra (Table S6 and Fig. 5). The other flatworm parasites constitute singletons (a, c, e, f, g, and h) in the phylogenetic trees and are here—together with clades b, i, and d—considered new species (see “Discussion”). The genetic similarities of the 18S rDNA sequences between all 16 species of *Rhytidocystis* in our trees, i.e., the nine new species a–i, *Rhytidocystis* sp. 1, *R. cyamus*, *R. polygordiae*, *R. dobrovolskiji*, *R. nekhoroshkovae*, *R. pertsovi*, and the unidentified species of *Rhytidocystis* from *Ostrea edulis* (BOU83331) varies between 53.76% and 95.53%.

### Cophylogenetic analyses between hosts and parasites

A tanglegram summarizing the cophylogeny between *Rhytidocystis* and their flatworm hosts reveals a complex association pattern suggesting a general incongruence between the two phylogenies (Fig. 5A). In addition, the null hypothesis that the host phylogeny does not constrain the parasite phylogeny in the Procrustean Approach to Cophylogeny (PACo) analysis cannot be rejected based on the goodness-of-fit statistic (*m*^*2*^_*XY*_ = 1.551) and its p-value (0.076).

The squared residuals and their 95% confidence intervals representing contributions of each individual host-parasite association to the global fit are shown in Fig. 5C. The host-parasite links of the proseriate (*Duplominona* sp.–*Rhytidocystis* QU51) and to a lesser extent those of the thalassotyphloplanids (*Parapharyngiella* n. sp.–*Rhytidocystis* CU259, *Parapharyngiella* n. sp.–*Rhytidocystis* CU205, *Trigonostomum vanmecheleni–Rhytidocystis* HO1) and schizorhynchids (*Carcharodorhynchus* n. sp. 1–*Rhytidocystis* OK10, *Carcharodorhynchus* n. sp. 2–*Rhytidocystis* CU271) have high squared residuals, thus contributing to the overall incongruence of the host and parasite phylogenies. These high values can be linked to the respective phylogenetic positions of the rhytidocystids from these hosts. For instance, *Rhytidocystis* QU51 from the proseriate *Duplominona* sp. is nested among rhytidocystids infecting rhabdocoels, while the rhytidocystids from the thalassotyphloplanids and schizorhynchids are spread over the different clades b, j and k, and clades b and k, respectively (Fig. 5A). Finally, *Rhytidocystis* CU260 is also nested in clade b, while its host, *Mariplanella piscadera*, is a representative of the most basal rhabdocoel clade Mariplanellida (see Fig. 4A). This also results in a value higher than the median for the squared residual of this host-parasite association.

The three individual host-parasite links of *Utelga heinckei* (*Utelga heinckei*–*Rhytidocystis* QU4, *Utelga heinckei*–*Rhytidocystis* QU43, *Utelga heinckei*–*Rhytidocystis* QU44) and those of all polycystidid flatworms (*Myobulla* sp.–*Rhytidocystis* CU255, *Myobulla* sp.–*Rhytidocystis* CU256, *Austrorhynchus hawaiiensis*–*Rhytidocystis* CU226, *Polycystidinae* sp.–*Rhytidocystis* CU1274) have relatively low squared residuals, indicating some phylogenetic congruence for these associations. Both hosts and parasites of these associations cluster together in their respective phylogenies (Fig. 5A). The low squared residual values for the *Cheliplana terminalis*–*Rhytidocystis* OK1, *Reinhardorhynchus riegeri*–*Rhytidocystis* CU1272, and *Parautelga* sp.–*Rhytidocystis* OK5 associations are harder to explain based on the phylogenetic positions of hosts and parasites in their respective phylogenies. Possibly the basal position of *Rhytidocystis* OK1 among rhytidocystids and the relatively basal position of cheliplanids among Kalyptorhynchia—the latter taxon comprising the majority of host microturbellarians in this study—contribute to the very low squared residual value of its corresponding host-parasite association.

## Discussion

### Rhytidocystids infect a diversity of host taxa around the world

The genus *Rhytidocystis* Henneguy, 1907 belongs to a poorly studied group of apicomplexans historically classified as agamococcidians. The order Agamococcidiorida Levine, 1979 was originally erected to accommodate coccidian-like apicomplexans with neither gametogony (sexual reproduction) nor merogony (asexual reproduction)^43^. Its two monotypic families, Rhytidocystidae Levine, 1979 and Gemmocystidae Upton & Peters, 1986, contained the genera *Rhytidocystis* and *Gemmocystis* Upton & Peters, 1986, respectively. All eight described species of *Rhytidocystis* infect the intestinal epithelium, coelom and connective tissues of polychaete annelids from the northeast and northwest Atlantic Ocean, the northeast Pacific Ocean and the Arctic Ocean^43–49^. The only known species of *Gemmocystis* is found in the mesenterial filaments of several species of scleractinian corals^50^. Recent phylogenetic and phylogenomic studies have confirmed the close affinity of rhytidocystids to other coccidiomorphs including coccidians and hematozoans^34,49,51^. In addition, Miroliubova et al. (2020) and Mathur et al. (2021) showed that rhytidocystids form a clade with different representatives of Aggregatidae and *Margolisiella*, which was subsequently named Eococcidia and Marosporida, respectively^34,49^. It is now thought that *Gemmocystis cylindricus* Upton & Peters, 1986 is likely a “type-N” apicomplexan belonging to the corralicolids^52–54^. As a result, the taxonomic validity of the agamococcidians has been questioned^34^.

Our results confirm that rhytidocystids are not confined to annelids, but are widespread among marine flatworms. Their host range is almost certainly wider as rhytidocystids have also been identified in the eukaryotic microbiome of microscopic molluscs and arthropods^5^. Moreover, the 18S rDNA sequence of an unidentified apicomplexan from the European oyster *Ostrea edulis* is represented in our tree as BOU83331 (Fig. 4C) and is clearly also a rhytidocystid^49^. The fact that all parasites in the microturbellarians collected over a span of six years in four geographically distinct locations are identified as *Rhytidocystis* strongly suggests that the historical records of “gregarines”^16–23^, “sporozoans”^24,25^, or “apicomplexans”^26^ observed in other marine rhabdocoels and proseriates around the world involve rhytidocystids as well. However, not all apicomplexan cells would easily be recognized within the bodies of their microturbellarian host. Rhytidocystid cells, for instance, could be mistaken for food items or be obscured by opaque parenchyma or be very similar in colour and blend in with the surrounding host tissues (see for instance Fig. 2D). In addition, invertebrate zoologists might lack the taxonomic training for recognizing these parasites altogether. Therefore, many infections could have gone unnoticed and would have not been reported in the microturbellarian or meiofaunal literature. For example, in a recent report on trigonostomid rhabdocoels from British Columbia, one of the specimens of *Trigonostomum tori* illustrated in Van Steenkiste and Leander (2018) is almost certainly parasitized by apicomplexans, but the cells were at first considered as food items^55^. Many of the flatworm host genera from this study, including *Carcharodorhynchus*, *Cheliplana*, *Trigonostomum*, *Austrorhynchus* and *Duplominona* have global distributions further indicating that we have only started uncovering a small fraction of the diversity of apicomplexan parasites associated with these animals.

*Rhytidocystis* was found to infect representatives of all major marine rhabdocoel lineages, except for the neodalyellids. Despite the collection of many members of this taxon around Vancouver Island^56^ and Japan (unpublished data), we did not observe any apicomplexans in this group of rhabdocoels. Interestingly, no mentions of “sporozoans” or “gregarines” from free-living neodalyellids could be found in the literature either. The only two records of apicomplexans in this group are both hyperparasites in endosymbiotic neodalyellids from the southern hemisphere, including *Monocystella epibatis* from an Australian pterastericolid^27^, and “sporozoans” from an Antarctic umagillid^57^.

Prevalence of these parasites in flatworm hosts, when recorded, seemed relatively low. For instance, Den Hartog (1964a) found “gregarines” in only one single individual of the rhabdocoel *Ptychopera westbladi* (Luther, 1943) out of the 300 individuals he observed from the Rhine-Meuse-Scheldt Delta in the Netherlands^16^. The same author also found the proseriate *Monocelis fusca* in great numbers around the same region, but states that he “rarely found parasitic gregarines in the body of *M. fusca*”^17^. This is in line with our quantitative observations in Japan, Canada and Curaçao.

### Undescribed species diversity within flatworms and rhytidocystids

The marine rhabdocoel diversity in the sampled regions has been reported in several studies featuring species and records from Curaçao^58–64^, British Columbia^55,56,62,65–67^, and Hokkaido^68^. No marine rhabdocoels are currently known from the Ryukyu Islands. *Duplominona* sp. is the first record of a proseriate from British Columbia, but several species of proseriates have been documented from the adjacent San Juan Islands, Washington^69,70^.

Some of the host species were only recently described, including *Mariplanella piscadera* and *Trigonostomum vanmecheleni*^63,71,72^, while others, including the two species of *Carcharodorhynchus* (Fig. 2B,E,F) and one species of *Parapharyngiella*, are new to science and await formal description. *Parautelga* sp. and *Myobulla* sp. could not be identified to species level and no other rDNA sequences of representatives of these genera were available in public databases for comparison; however, they still fell within the expected higher-level taxa Typhlopolycystidinae (Polycystididae) and Koinocystididae, respectively (Fig. 4A). Additional morphological data are also needed for *Duplimona* sp. to establish whether or not this species is new to science. *Polycystidinae* sp. could not be identified to genus level because its internal morphology was obscured by dark pigment. Based on its phylogenetic position (Fig. 4A), it seems closely related to an assemblage of genera that were formerly classified within different polycystidid subfamilies^73^.

Several lines of evidence suggest that at least nine new species of *Rhytidocystis* can be recognized in our flatworm hosts. These lines of evidence include (1) the presence of distinct clades and lineages of 18S rDNA sequences in our phylogenetic trees (Fig. 5A), (2) a barcode gap of at least 3.52% 18S rDNA sequence variation between the species (Table S6 and Fig. S1), (3) geographical distributions, (4) host associations, and (5) ultrastructural differences on the cell surface of the four cells we were able to characterize with SEM (Figs. 3, 5, and Tables S1 and S4). Although we could establish these new species using primarily molecular data and host associations, future studies that focus on characterizing the diagnostic morphological characters (if available) of each of these flatworm-infecting species will allow for their formal description and taxonomic designation.

### Complicated patterns of host specificity, phylosymbiosis and biogeography

Our results paint a complex picture pertaining to host specificity and patterns of phylosymbiosis between the flatworm hosts and their rhytidocystid parasites. While our cophylogenetic analyses show an overall incongruence between host and parasite phylogenies, several other patterns can be discerned.

First, the same host species can be infected by more than one putative species of *Rhytidocystis* as is the case for the thalassotyphloplanid flatworm *Parapharyngiella* n. sp. The two infected specimens of this flatworm were collected in roughly the same location (Piscadera bay, Curaçao, Table S4). This particular location in Curaçao seems to be a hotspot for microturbellarian and parasite diversity with three different putative species of *Rhytidocystis* infecting seven species of rhabdocoel flatworms belonging to several higher-level taxa. The other (sub)tropical sampling locations on Okinawa were also relatively diverse with three putative species of *Rhytidocystis* infecting three different rhabdocoel taxa. A variety of sampling locations and marine habitats were surveyed for microturbellarians on Curaçao and Okinawa, but only locations with detritus-enriched coral sand seemed to harbour infected hosts.

Some species of *Rhytidocystis* can infect various flatworm host species. Species ‘b’ infects hosts belonging to the three major groups of rhabdocoels (Mariplanellida, Kalyptorhynchia and Dalytyphloplanida)^63^, while species ‘i’ was found in three different species of Polycystididae, one of the most species-rich families of kalyptorhynchs. This suggests varying degrees of host specificity among species of rhytidocystids infecting rhabdocoels—species ‘b’ in any rhabdocoel versus species ‘i’ only in polycystidids—but larger datasets are needed to confirm this trend.

All of our sampling locations are characterized by a relatively high diversity of microturbellarians and other meiofaunal organisms: there were at least eight and four different species of rhabdocoels in the samples from Onna Bay and Igei Beach (Okinawa), respectively; fifteen species of rhabdocoels from Piscadera Bay (Curaçao); seven species of epiphytic rhabdocoels from Otaru (Hokkaido); and no less than fifteen species of rhabdocoels and three species of proseriates from Hyacinthe Bay on Quadra Island. Only some of these species were infected with rhytidocystids, including seven species from Piscadera Bay (Curaçao), three species from Onna Bay (Okinawa), two species from Igei Beach (Okinawa), one species from Otaru (Hokkaido), and one species from Hyacinthe bay (Quadra). As such, species of *Rhytidocystis* with relatively wide host ranges could have been present in several other host species, but probably require the collection of sufficient numbers of individuals for each host species to be found. The rhytidocystids present in an unidentified species of *Reinhardorhynchus* (Koinocystididae) and an unidentified species of *Cheliplana* (Cheliplanidae) from Onna Bay (Okinawa) could not be characterized with molecular and/or morphological data in this study.

Finally, *Rhytidocystis* diversity and perceived prevalence are higher in Curaçao and Okinawa (two and one sampling campaign, respectively) compared to Quadra Island (repeated seasonal surveys since 2021), suggesting that the diversity and prevalence of these parasites might be influenced by geography—i.e., higher diversity and prevalence in (sub)tropical marine areas compared to cooler temperate marine areas. In addition, our results also show the potential existence of a (sub)tropical *Rhytidocystis* species group (clade k) and a species group (clade j) inhabiting cooler temperate seas (Fig. 5A,B). Additional collections in other sampling locations around the world are needed to confirm these geographical links within a molecular phylogenetic context.

### Supplementary Information

Supplementary Information.

## Data Availability

Genbank accession numbers of the sequences generated for this study are included in Tables S3 and S4.
