# Supplementary material for: Patterns of host-parasite associations between marine meiofaunal flatworms (Platyhelminthes) and rhytidocystids (Apicomplexa)
Source: Sci Rep. 2023 Nov 29;13:21050. doi: 10.1038/s41598-023-48233-y (PMC10687266; doi:10.1038/s41598-023-48233-y)

**Supporting information**

**Table S1.** Collection data of the flatworm hosts containing apicomplexan parasites.

| Host | Location and date | Coordinates | Habitat |
| --- | --- | --- | --- |
| RHABDOCOELA |  |  |  |
| *MARIPLANELLIDA* |  |  |  |
| *Mariplanella piscadera* | Piscadera Bay, Curaçao (April 2018) | 12°07′17.5"N 68°58′09.4"W | Coarse, detritus-rich sand, 2.5 m deep |
| *KALYPTORHYNCHIA* |  |  |  |
| Schizorhynchia |  |  |  |
| *Cheliplana terminalis* | Igei, Okinawa, Japan (February 2019) | 26°27'17.9"N 127°52'27.5"E | Medium, detritus-rich sand, intertidal |
| *Carcharodorhynchus* n. sp.1 | Onna, Okinawa, Japan (February 2019) | 26°29'05.1"N 127°50'25.6"E | Coarse coral sand from seagrass bed, intertidal |
| *Carcharodorhynchus* n. sp.2 | Piscadera Bay, Curaçao (April 2018) | 12°07′17.5"N 68°58′09.4"W | Coarse, detritus-rich sand, 2.5 m deep |
| Koinocystididae |  |  |  |
| *Reinhardorhynchus riegeri* | Piscadera Bay, Curaçao (April 2018) | 12°07'19.6"N 68°58'09.8"W | Coarse, detritus-rich sand, 3 m deep |
| *Parautelga* sp. | Igei, Okinawa, Japan (February 2019) | 26°27'17.9"N 127°52'27.5"E | Medium, detritus-rich sand, intertidal |
| *Utelga heinckei* | Hyacinthe Bay, Quadra, BC, Canada (September 2021) | 50°07'01.6"N 125°13'21.8"W | Fine, detritus-rich sand, 9 m deep |
| *Utelga heinckei* | Hyacinthe Bay, Quadra, BC, Canada (January 2022) | 50°06'59.5"N 125°13'22.6"W | Medium, detritus-rich sand from seagrass bed, 5.2 m deep |
| *Utelga heinckei* | Hyacinthe Bay, Quadra, BC, Canada (January 2022) | 50°06'59.5"N 125°13'22.6"W | Medium, detritus-rich sand from seagrass bed, 5.2 m deep |
| Polycystididae |  |  |  |
| *Myobulla* sp. | Piscadera Bay, Curaçao (April 2018) | 12°07′17.5"N 68°58′09.4"W | Coarse, detritus-rich sand, 2.5 m deep |
| *Myobulla* sp. | Piscadera Bay, Curaçao (April 2018) | 12°07′17.5"N 68°58′09.4"W | Coarse, detritus-rich sand, 2.5 m deep |
| *Polycystidinae* sp. (unidentified genus) | Piscadera Bay, Curaçao (April 2018) | 12°07'19.6"N 68°58'09.8"W | Coarse, detritus-rich sand, 3 m deep |
| *Austrorhynchus hawaiiensis* | Piscadera Bay, Curaçao (April 2016) | 12°07'19.6"N 68°58'09.8"W | Coarse, detritus-rich sand, 3 m deep |
| *DALYTYPHLOPLANIDA* |  |  |  |
| Thalassotyphloplanida |  |  |  |
| *Parapharyngiella* n. sp. | Piscadera Bay, Curaçao (April 2016) | 12°07'19.6"N 68°58'09.8"W | Coarse, detritus-rich sand, 3 m deep |
| *Parapharyngiella* n. sp. | Piscadera Bay, Curaçao (April 2018) | 12°07′17.5"N 68°58′09.4"W | Coarse, detritus-rich sand, 2.5 m deep |
| *Trigonostomum vanmecheleni* | Otaru, Hokkaido, Japan (February, 2019) | 43°13'31.2"N 141°01'04.0"E | Macro-algae, intertidal |
| PROSERIATA |  |  |  |
| Monocelididae |  |  |  |
| *Duplominona* sp. | Hyacinthe Bay, Quadra, BC, Canada (January 2022) | 50°07'01.6"N 125°13'21.8"W | Fine, detritus-rich sand, 9 m deep |
|  | | |  |

**Table S2.** Amplification primers, internal sequencing primers and PCR thermocycling conditions.

|  | Forward primers (sequence) | Reverse primers (sequence) |
| --- | --- | --- |
| HOST |  |  |
| 18S amplification and sequencing | TimA (5’-AMCTGGTTGATCCTGCCAG-3’) | TimB (5’-TGATCCATCTGCAGGTTCACCT-3’) |
|  | TimA (5’-AMCTGGTTGATCCTGCCAG-3’) | Poly18SR2 (5’-GCMRGKTCACCTACRGAAACCTTGTT-3’) |
|  | TimA (5’-AMCTGGTTGATCCTGCCAG-3’) | R18SR (5’-GTTCACCTACGGAAACCTTGTT-3’) |
| 18S sequencing only | 600F (5’-GGTGCCAGCAGCCGCGGT-3’) | 600R (5’-ACCGCGGCTGCTGGCACC-3’) |
|  | 1100F (5’-CAGAGGTTCGAAGACGATC-3’) | 1100R (5’-GATCGTCTTCGAACCTCTG-3’) |
|  | 18S7F (5’-GCAATAACAGGTCTGTGATGC-3’) | 18S7FK (5’-GCATCACAGACCTGTTATTGC-3’) |
| 28S amplification and sequencing | LSU5 (5’-TAGGTCGACCCGCTGAAYTTA-3’) | LSUD6.3B (5’-GCTGTTCACATGGAACCCTTCTC-3’) |
| 28S sequencing only | L300F (5’-CAAGTACCGTGAGGGAAAGTTG-3’) | L300R (5’-CAACTTTCCCTCACGGTACTTG-3’) |
|  | L1200F (5’-CCCGAAAGATGGTGAACTATG-3’) | L1200R (5’-GCATAGTTCACCATCTTTCGG-3’) |
| PARASITE |  |  |
| 18S amplification and sequencing | RhyIntF1 (5’-GAAGACTGTGGATAGCTCAT-3’) – Internal | RhyIntR2 (5’-CTTCTCCTTCCTCTAARTGATAAG-3’) – External |
|  | TimA (5’-AMCTGGTTGATCCTGCCAG-3’) – External | Rh1150R (5’-CTTTGATTTCTCATAAGGTTCTG-3’) – Internal |
|  | Rh1100F (5’-TAAACTATGCCRACTAGAGATTG-3’) – Internal | R18SR (5’-GTTCACCTACGGAAACCTTGTT-3’) – Internal |
|  | 1100F (5’-CAGAGGTTCGAAGACGATC-3’) – Internal | Poly18SR2 (5’-GCMRGKTCACCTACRGAAACCTTGTT-3’) – External/Internal |
| 18S sequencing | 600F (5’-GGTGCCAGCAGCCGCGGT-3’) – Internal | 600R (5’-ACCGCGGCTGCTGGCACC-3’) – Internal |
| Thermocycling:  Touchdown PCR: 95°C for 3m, touch down in 9 cycles (94°C for 30s, 60°C down to 56°C for 30s, 72°C for 1m30s), 27­–39 cycles (94°C for 30s, 55°C for 30s, 72°C for 1m30s), 72°C for 5m  Nested PCR Initial Reaction (With External Primers): 94°C for 1m, 35 cycles (94°C for 30s, 52°C for 5s, 68°C for 10s), 68°C for 5m  Nested PCR Second Reaction (With Internal Primers): 94°C for 1m, 35 cycles (94°C for 30s, 52°C for 5s, 68°C for 10s), 68°C for 5m | | |

**Table S3.** 18S and 28S GenBank accession numbers of the flatworm taxa used in the phylogenetic analyses. The host taxa harbouring *Rhytidocystis* endosymbionts are in bold. Amplicon sizes of novel sequences are between parentheses.

| Taxon | 18S | 28S |
| --- | --- | --- |
| RHABDOCOELA |  |  |
| *MARIPLANELLIDA* |  |  |
| *Mariplanella frisia* | AJ012514 | – |
| *Mariplanella piscadera* (CU260) | **OM339545** | **OM339542** |
| *KALYPTORHYNCHIA* |  |  |
| *Toia* sp. 1 | OM339546 | OM339543 |
| *Toia* sp. 2 | OM339547 | OM339544 |
| *Toia ycia* | KC869828 | KC869881 |
| Schizorhynchia |  |  |
| *Cheliplanilla caudata* | KJ887449 | KJ887502 |
| *Cheliplana* sp.3 | KR339024 | – |
| *Cheliplana* cf. *orthocirra* | AJ012507 | – |
| *Cheliplana terminalis* (OK273) | **OR490852 (1765 bp)** | **OR490868 (1673 bp)** |
| *Cheliplana* sp.1 | KR339022 | KR339049 |
| *Cheliplana* sp.2 | KR339023 | KR339050 |
| *Proschizorhynchus tricingulatus* | KJ887423 | KJ887503 |
| *Thylacorhynchus conglobatus* | KJ887448 | KJ887534 |
| *Carcharodorhynchus* sp.1 | KR339017 | – |
| *Carcharodorhynchus* sp.2 | KR339018 | KR339047 |
| *Carcharodorhynchus* sp.3 | KR339019 | – |
| *Carcharodorhynchus* sp.4 | KR339020 | – |
| *Carcharodorhynchus flavidus* | KJ887457 | KJ887563 |
| *Carcharodorhynchus* n. sp.1 (OK293) | **OR490851 (1786 bp)** | **OR490867 (1731 bp)** |
| *Carcharodorhynchus* n. sp.2 (CU271) | **OR490850 (2043 bp)** | **OR490866 (1821 bp)** |
| Cicerinidae |  |  |
| *Acrumena massiliensis* | KJ887417 | KJ887509 |
| *Cicerina tetradactyla* | KJ887465 | KJ887520 |
| *Ptyalorhynchus coecus* | KJ887416 | KJ887550 |
| Placorhynchidae |  |  |
| *Placorhynchus dimorphis* | KJ887409 | KJ887507 |
| Zonorhynchidae |  |  |
| *Zonorhynchus tvaerminnensis* | KJ887455 | KJ887516 |
| Psammorhynchidae |  |  |
| *Psammorhynchus tubulipenis* | KJ887438 | KJ887561 |
| Gnathorhynchidae |  |  |
| *Gnathorhynchus inermis* | KJ887402 | KJ887524 |
| Cystiplanidae |  |  |
| *Cystiplex axi* | KJ887437 | KJ887549 |
| *Cystiplex* sp. | KJ887469 | KJ887495 |
| Koinocystididae |  |  |
| *Koinogladius sinensis* | MF443159 | MF443174 |
| *Rhinolasius dillonicus* | MW081602 | MW054461 |
| *Reinhardorhynchus tahitiensis* | MW054462 | MW054453 |
| *Reinhardorhynchus riegeri* | MW081595 | MW054454 |
| *Reinhardorhynchus riegeri* (CU1272) | **OR490859 (1747 bp)** | **OR490875 (1672 bp)** |
| *Reinhardorhynchus anamariae* | MW081597 | MW054456 |
| *Itaipusa* sp.1 | KJ887451 | KJ887557 |
| *Itaipusa karlingi* | MW081598 | MW054457 |
| *Itaipusa biglandula* | MW081601 | MW054460 |
| *Itaipusa divae* | MW081596 | MW054455 |
| *Itaipusa novacaledonica* | KJ887481 | KJ887528 |
| *Utelga pseudoheinckei* | – | MW054458 |
| *Utelga* sp. p | MW081599 | – |
| *Parautelga* sp. (OK280) | **OR490858 (1761 bp)** | **OR490874 (1667 bp)** |
| *Utelga heinckei* (QU4) | **OR490861 (1764 bp)** | **OR490876 (1667 bp)** |
| *Utelga heinckei* (QU43) | **OR490862 (1764 bp)** | – |
| *Utelga heinckei* (QU44) | **OR490863 (1764 bp)** | **OR490877 (1667 bp)** |
| *Utelga* sp. h | MW081600 | – |
| *Utelga heinckei* | – | MW054459 |
| Polycystididae |  |  |
| *Brachyrhynchoides triplostylis* | KJ887399 | KJ887558 |
| *Djeziraia euxinica* | KJ887442 | KJ887527 |
| *Limipolycystis wallbergi* | KJ887467 | KJ887491 |
| *Brunetorhynchus canariensis* | KJ887474 | KJ887485 |
| *Brunetorhynchus microstylis* | KJ887468 | KJ887494 |
| *Myobulla* sp. (CU255) | **OR490854 (1767 bp)** | **OR490870 (1685 bp)** |
| *Myobulla* sp. (CU256) | **OR490855 (1767 bp)** | **OR490871 (1661 bp)** |
| *Lagenopolycystis* sp.1 | KJ887403 | KJ887569 |
| *Lagenopolycystis mandelai* | KJ887441 | KJ887536 |
| *Lagenopolycystis* sp.3 | KJ887434 | KJ887518 |
| *Lagenopolycystis peresi* | KJ887452 | – |
| *Phonorhynchella biarcuata* | KJ887447 | KJ887548 |
| *Gallorhynchus mediterraneus* | KJ887428 | KJ887496 |
| *Gyratrix proaviformis* | KJ887430 | KJ887565 |
| *Scanorhynchus forcipatus* | KJ887412 | KJ887556 |
| *Opisthocystis goettei* | KJ887445 | KJ887559 |
| *Phonorhynchus helgolandicus* | KJ887478 | KJ887501 |
| *Progyrator mamertinus* | KJ887401 | KJ887493 |
| *Duplacrorhynchus megalophallus* | KJ887443 | KJ887535 |
| *Polycystidinae* sp. (unidentified genus) (CU1274) | **OR490853 (1751 bp)** | **OR490869 (1666 bp)** |
| *Acrorhynchides robustus* | KJ887458 | KJ887517 |
| *Paulodora drepanophora* | KJ887482 | KJ887544 |
| *Rogneda reticulata* | KJ887479 | KJ887529 |
| *Austrorhynchus bruneti* | KJ887405 | KJ887498 |
| *Austrorhynchus* sp.1 | KJ887431 | KJ887492 |
| *Austrorhynchus* sp.2 | KJ887480 | KJ887506 |
| *Austrorhynchus hawaiiensis* (CU226) | **OR490849 (1771 bp)** | **OR490865 (1660 bp)** |
| *Austrorhynchus hawaiiensis* | KJ887400 | KJ887572 |
| *DALYTYPHLOPLANIDA* |  |  |
| Thalassotyphloplanida |  |  |
| *Kytorhynchidae* sp. 1 | KC529401 | KC529527 |
| *Kytorhynchus* sp. | KC529400 | KC529526 |
| *Coronhelmis multispinosus* | KC529427 | KC529555 |
| *Cilionema hawaiiensis* | KC529428 | KC529556 |
| *Parapharyngiella* sp. | KC529405 | KC529531 |
| *Parapharyngiella* n. sp. | MF321750 | – |
| *Parapharyngiella* n. sp. (CU205) | **OR490856 (1828 bp)** | **OR490872 (1783 bp)** |
| *Parapharyngiella* n. sp. (CU259) | **OR490857 (1843 bp)** | **OR490873 (1784 bp)** |
| *Ptychopera japonica* | MF321751 | MF321760 |
| *Trigonostomum penicillatum* | KC529414 | KC529540 |
| *Trigonostomum venenosum* | KC529417 | KC529543 |
| *Trigonostomum watsoni* | KC529415 | KC529541 |
| *Trigonostomum denhartogi* | AY775773 | – |
| *Trigonostomum tillicum* | MF321753 | MF321762 |
| *Trigonostomum vanmecheleni* (HO315) | **OR490860 (1785 bp)** | – |
| *Trigonostomum vanmecheleni* | MN316548 | MN316544 |
| *Trigonostomum vanmecheleni* | MN316549 | MN316545 |
| *Trigonostomum armatum* | KC529419 | KC529545 |
| *Trigonostomum franki* | KC529416 | KC529542 |
| *Trigonostomum setigerum* | KC529418 | KC529544 |
| *Trigonostomum tori* | MF321754 | MF321763 |
| *Ceratopera gracilis* | KC529422 | KC529549 |
| *Beklemischeviella angustior* | KC529412 | KC529538 |
| *Proxenetes quinquespinosus* | KC529406 | KC529532 |
| *Promesostoma caligulatum* | KC529432 | KC529560 |
| *Microvahine corallicola* | KC529423 | KC529550 |
| *Thalassoplanella collaris* | KC529483 | KC529614 |
| *Brinkmanniella palmata* | KC529424 | KC529553 |
| Limnotyphloplanida |  |  |
| *Halammovortex* sp. | KC529437 | KC529567 |
| *Scutariella sinensis* | MF773690 | MF773687 |
| *Temnosewellia minor* | AY157183 | AY157164 |
| *Castrella truncate* | KC529439 | KC529570 |
| *Gieysztoria rubra* | KC529480 | KC529611 |
| *Pseudodalyellia alabamensis* | KC529440 | KC529571 |
| *Dalyellia viridis* | KC529444 | KC529575 |
| *Microdalyellia fusca* | KC529453 | KC529584 |
| *Acrochordonoposthia conica* | KC529487 | KC529617 |
| *Opistomum arsenii* | KC529491 | KC529620 |
| *Typhloplana viridata* | KC529484 | KC529615 |
| *Bryoplana xerophila* | KC529489 | KC529619 |
| *Phaenocora foliacea* | KC529492 | KC529621 |
| *Strongylostoma radiatum* | KC529485 | KC529616 |
| Neodalyellida |  |  |
| *Adenopharynx mitrabursalis* | KC529520 | KC529641 |
| *Wahlia macrostylifera* | KC529518 | KC529639 |
| *Tamanawas kalipis* | MH337259 | MH337262 |
| *Bresslauilla relicta* | KC869832 | KC869885 |
| *Balgetia semicirculifera* | KC529503 | KC529628 |
| *Pogaina sinensis* | MK509001 | MK509007 |
| *Baicalellia canadensis* | KC869833 | KC869886 |
| *Pterastericola psilastericola* | KC529516 | KC529637 |
| PROSERIATA |  |  |
| Ciliopharyngiellidae |  |  |
| *Ciliopharyngiella constricta* | AY775754 | – |
| *Ciliopharyngiella constricta* | AY775755 | – |
| Unguiphora |  |  |
| *Nematoplana coelogynoporoides* | KJ682383 | KJ682445 |
| *Nematoplana* sp. | AJ270160 | AJ270175 |
| *Polystyliphora novaehollandiae* | AJ270161 | AJ270177 |
| *Polystyliphora filum* | KY320104 | KY320164 |
| *Polystyliphora karlingi* | KC869815 | KC869868 |
| Calviriidae |  |  |
| *Calviria solaris* | AJ270153 | AJ270168 |
| Coelogynoporidae |  |  |
| *Parainvenusta englarorum* | HM026563 | HM026556 |
| *Vannuccia campana* | HM026566 | HM026559 |
| *Cirrifera cirrifera* | HM026564 | HM026557 |
| *Coelogynopora gynocotyla* | AJ243679 | AJ270170 |
| *Invenusta aestus* | HM026565 | HM026558 |
| Otoplanidae |  |  |
| *Notocaryoplana arctica* | HM026568 | HM026561 |
| *Parotoplana renatae* | AJ012517 | AJ270176 |
| *Archotoplana holotricha* | AJ243676 | AJ270165 |
| *Xenotoplana acus* | AJ270155 | AJ270181 |
| Archimonocelididae |  |  |
| *Archimonocelis staresoi* | AJ270152 | AJ270166 |
| *Archimonocelis crucifera* | AJ270151 | AJ270163 |
| Monocelididae |  |  |
| *Monotoplana* cf. *diorchis* | AJ270159 | AJ270174 |
| *Monocelis longistyla* | KR364618 | KR364663 |
| *Minona ileanae* | JN224905 | JN224910 |
| *Pseudomonocelis cetinae* | JN224899 | JN224913 |
| *Duplominona brasiliensis* | KJ682367 | KJ682429 |
| *Duplominona* sp. (QU51) | **OR490864 (1764 bp)** | – |
| *Duplominona tridens* | KJ682371 | KJ682433 |
|  | | |

**Table S4.** 18S GenBank accession numbers and amplicon sizes (between parentheses) of the new rhytidocystids from Curaçao, Hokkaido, Okinawa and Quadra Island. See Table S1 for detailed metadata of the hosts.

| Parasite | Host | Location and date | 18S |
| --- | --- | --- | --- |
| *Rhytidocystis* OK1 | *Cheliplana terminalis* | Igei, Okinawa, Japan (February 2019) | OR496156 (1702 bp) |
| *Rhytidocystis* CU226 | *Austrorhynchus hawaiiensis* | Piscadera Bay, Curaçao (April 2016) | OR496153 (1593 bp) |
| *Rhytidocystis* CU255 | *Myobulla* sp. | Piscadera Bay, Curaçao (April 2018) | OR496160 (1700 bp) |
| *Rhytidocystis* CU256 | *Myobulla* sp. | Piscadera Bay, Curaçao (April 2018) | OR496161 (1593 bp) |
| *Rhytidocystis* CU1274 | *Polycystidinae* sp. | Piscadera Bay, Curaçao (April 2018) | OR496158 (1593 bp) |
| *Rhytidocystis* QU51 | *Duplominona* sp. | Hyacinthe Bay, Quadra, BC, Canada (January 2022) | OR496157 (1683 bp) |
| *Rhytidocystis* OK5 | *Parautelga* sp. | Igei, Okinawa, Japan (February 2019) | OR496164 (1622 bp) |
| *Rhytidocystis* CU205 | *Parapharyngiella* n. sp. | Piscadera Bay, Curaçao (April 2016) | OR496162 (1154 bp) |
| *Rhytidocystis* OK10 | *Carcharodorhynchus* n. sp.1 | Onna, Okinawa, Japan (February 2019) | OR496155 (1674 bp) |
| *Rhytidocystis* HO1 | *Trigonostomum vanmecheleni* | Otaru, Hokkaido, Japan (February, 2019) | OR496166 (1641 bp) |
| *Rhytidocystis* QU44 | *Utelga heinckei* | Hyacinthe Bay, Quadra, BC, Canada (January 2022) | OR496169 (1679 bp) |
| *Rhytidocystis* QU4 | *Utelga heinckei* | Hyacinthe Bay, Quadra, BC, Canada (September 2021) | OR496168 (1599 bp) |
| *Rhytidocystis* QU43 | *Utelga heinckei* | Hyacinthe Bay, Quadra, BC, Canada (January 2022) | OR496167 (1599 bp) |
| *Rhytidocystis* CU271 | *Carcharodorhynchus* n. sp.2 | Piscadera Bay, Curaçao (April 2018) | OR496154 (1708 bp) |
| *Rhytidocystis* CU260 | *Mariplanella piscadera* | Piscadera Bay, Curaçao (April 2018) | OR496159 (1708 bp) |
| *Rhytidocystis* CU1272 | *Reinhardorhynchus riegeri* | Piscadera Bay, Curaçao (April 2018) | OR496165 (1600 bp) |
| *Rhytidocystis* CU259 | *Parapharyngiella* n. sp. | Piscadera Bay, Curaçao (April 2018) | OR496163 (1708 bp) |

**Table S5.** 18S and 28S GenBank accession numbers of all other squirmid, chrompodellid and apicomplexan taxa used in the analyses in order to establish the phylogenetic position of the new rhytidocystids.

| Taxon | 18S | 28S |
| --- | --- | --- |
| SQUIRMIDA |  |  |
| *Platyproteum vivax* | AY196708 | – |
| *Platyproteum noduliferae* | LC663666 | LC663666 |
| CHROMPODELLIDA |  |  |
| *Colpodella angusta* | KU159286 | KU159286 |
| *Colpodella tetrahymenae* | AF330214 | – |
| *Voromonas pontica* | AF280076 | KU159287 |
| GREGARINES |  |  |
| *Ancora sagittata* | KX982503 | KX982503 |
| *Ascogregarina taiwanensis* | EF666482 | EF666482.1 |
| *Cephaloidophora* cf. *communis* | HQ891113 | HQ891113 |
| *Gregarina* sp. | JF412715 | JF412715 |
| *Heliospora* cf. *longissima* | HQ891114 | HQ891114 |
| *Lankesteria herdmaniae* | KR024697 | – |
| *Lankesteria metandrocarpae* | KR024699 | – |
| *Lecudina* cf. *tuzetae* | JF264860.1 | – |
| *Lecudina polymorpha* | FJ832158 | – |
| *Monocystis agilis* | AF457127 | – |
| *Neogregarinorida sp.* | AB748927 | AB748927 |
| *Polyrhabdina sp.* | MT214481 | MT214481 |
| *Pterospora schizosoma* | DQ093793 | – |
| *Selenidium planusae* | MN381958 | MN381958 |
| *Selenidium pygospionis* | MH061278 | MH061278 |
| CRYPTOSPORIDIUM |  |  |
| *Cryptosporidium hominis* | MN661184 | JIBM01000067 |
| *Cryptosporidium muris* | AB089284 | – |
| *Cryptosporidium parvum* | AF040725 | AF040725 |
| HEMATOZOA |  |  |
| *Babesia microti* | AB190287 | XR001160979 |
| *Plasmodium chabaudi* | XR007844653 | XR001678647 |
| *Plasmodium vivax* | XR003001225 | XR003001218 |
| *Theileria orientalis* | AB520958 | XR_696410 |
| *Theileria parva* | L02366 | U03602 |
| PROTOCOCCIDIA |  |  |
| *Eleutheroschizon duboscqi* | SRR9888047 | – |
| CORALLICOLIDA |  |  |
| *Anthozoaphila gnarlus* | MW192638 | MW192642 |
| *Corallicola aquarius* | MH304758 | MH304758 |
| EUCOCCIDIA |  |  |
| *Cystoisospora felis* | L76471 | U85705 |
| *Eimeria acervulina* | FJ236372 | GU593707 |
| *Goussia balatonica* | GU479650 | GU593717 |
| *Goussia desseri* | GU479641 | GU593705 |
| *Hammondia hammondi* | AH008381 | AF101077 |
| *Isospora butcherae* | KY801685 | KY801686 |
| *Sarcocystis zamani* | KU244524 | KU244524 |
| *Toxoplasma gondii* | XR001974458 | – |
| MAROSPORIDA |  |  |
| *Margolisiella islandica* | JN227668 | – |
| *Pseudoklossia pectinis* | MH348778 | – |
| *Rhytidocystis cyamus* | GQ149767 | – |
| *Rhytidocystis dobvovolskiji* | MT231947 | – |
| *Rhytidocystis nekhoroshkovae* | MT231950 | – |
| *Rhytidocystis pertsovi* | SRR9888045 | – |
| *Rhytidocystis pertsovi* | MT231948 | – |
| *Rhytidocystis polygordiae* | DQ273988 | – |
| *Rhytidocystis* sp. 1 | SRR9888046 | – |
| uncultured eukaryote KT072247 | KT072247 | – |
| uncultured eukaryote GU823445 | GU823445 | – |
| unidentified apicomplexan AB000912 ex. *Tridacna* sp. | AB000912 | – |
| unidentified apicomplexan CAM71 ex. *Mytilus edulis* | MN148114 | – |
| unidentified protist BOU83331 ex. *Ostrea edulis* | BOU83331 | – |
|  | | |

**Table S6.** Pairwise genetic similarities between the 25 18S rDNA sequences of the rhytidocystids used in the phylogenetic analyses. The nine putative species infecting microturbellarians are indicated with coloured borders and letters corresponding with the clades in Fig. 5A. The lowest and highest % identity (i.e., genetic similarity ranges) within species and among species are indicated with a grey background.

|  | RhCU226 | RhCU255 | RhCU256 | RhCU1274 | RhCU271 | RhCU260 | RhCU259 | RhCU1272 | RhCU205 | RhHO1 | RhQU43 | RhQU4 | RhQU44 | RhQU51 | RhOK10 | Rh_cya | Rh_sp1 | Rh_poly | RhOK5 | Rh_dob | Rh_nek | Rh_per | Rh_sp2 | BOU83331 | RhOK1 |
| --- | --- | --- | --- | --- | --- | --- | --- | --- | --- | --- | --- | --- | --- | --- | --- | --- | --- | --- | --- | --- | --- | --- | --- | --- | --- |
| RhCU226 | **i** | 99.968 | 99.903 | 99.578 | 83.785 | 83.699 | 83.796 | 83.731 | 56.833 | 84.722 | 84.787 | 84.787 | 84.787 | 84.787 | 84.409 | 85.71 | 83.281 | 85.229 | 82.537 | 80.527 | 79.399 | 81.592 | 81.624 | 80.467 | 80.632 |
| RhCU255 | 99.968 |  | 99.935 | 99.611 | 83.818 | 83.731 | 83.828 | 83.764 | 56.865 | 84.755 | 84.819 | 84.819 | 84.819 | 84.819 | 84.442 | 85.742 | 83.312 | 85.262 | 82.569 | 80.559 | 79.43 | 81.624 | 81.656 | 80.499 | 80.664 |
| RhCU256 | 99.903 | 99.935 |  | 99.546 | 83.753 | 83.667 | 83.764 | 83.699 | 56.801 | 84.69 | 84.755 | 84.755 | 84.755 | 84.755 | 84.377 | 85.677 | 83.249 | 85.197 | 82.505 | 80.496 | 79.367 | 81.561 | 81.592 | 80.435 | 80.6 |
| RhCU1274 | 99.578 | 99.611 | 99.546 |  | 83.495 | 83.409 | 83.505 | 83.441 | 56.541 | 84.432 | 84.496 | 84.496 | 84.496 | 84.496 | 84.119 | 85.419 | 82.997 | 84.939 | 82.247 | 80.241 | 79.051 | 81.306 | 81.338 | 80.243 | 80.408 |
| RhCU271 | 83.785 | 83.818 | 83.753 | 83.495 | **b** | 99.116 | 99.181 | 99.051 | 59.961 | 91.225 | 92.518 | 92.518 | 92.518 | 89.291 | 87.417 | 87.505 | 84.854 | 86.179 | 85.283 | 79.996 | 79.882 | 81.156 | 81.156 | 79.587 | 80.238 |
| RhCU260 | 83.699 | 83.731 | 83.667 | 83.409 | 99.116 |  | 99.871 | 99.871 | 59.702 | 90.88 | 92.238 | 92.238 | 92.238 | 89.205 | 87.33 | 87.355 | 84.833 | 86.028 | 85.133 | 80.038 | 79.861 | 81.071 | 81.071 | 79.821 | 80.217 |
| RhCU259 | 83.796 | 83.828 | 83.764 | 83.505 | 99.181 | 99.871 |  | 99.806 | 59.767 | 90.977 | 92.335 | 92.335 | 92.335 | 89.237 | 87.33 | 87.419 | 84.928 | 86.028 | 85.197 | 80.006 | 79.829 | 81.103 | 81.103 | 79.757 | 80.185 |
| RhCU1272 | 83.731 | 83.764 | 83.699 | 83.441 | 99.051 | 99.871 | 99.806 |  | 59.67 | 90.815 | 92.173 | 92.173 | 92.173 | 89.14 | 87.298 | 87.258 | 84.802 | 85.964 | 85.068 | 80.038 | 79.861 | 81.071 | 81.071 | 79.757 | 80.217 |
| RhCU205 | 56.833 | 56.865 | 56.801 | 56.541 | 59.961 | 59.702 | 59.767 | 59.67 | **e** | 59.871 | 59.612 | 59.612 | 59.612 | 59.585 | 60.388 | 58.527 | 56.207 | 57.393 | 57.578 | **53.763** | 53.768 | 54.933 | 54.965 | 54.068 | 54.692 |
| RhHO1 | 84.722 | 84.755 | 84.69 | 84.432 | 91.225 | 90.88 | 90.977 | 90.815 | 59.871 | **c** | 95.534 | 95.534 | 95.534 | 91.138 | 88.551 | 89.671 | 86.083 | 86.472 | 86.611 | 80.801 | 81.127 | 82.122 | 82.091 | 80.895 | 81.557 |
| RhQU43 | 84.787 | 84.819 | 84.755 | 84.496 | 92.518 | 92.238 | 92.335 | 92.173 | 59.612 | 95.534 | **d** | 100 | 100 | 91.462 | 88.551 | 89.154 | 85.957 | 86.667 | 86.481 | 80.992 | 81.127 | 82.569 | 82.473 | 80.895 | 81.174 |
| RhQU4 | 84.787 | 84.819 | 84.755 | 84.496 | 92.518 | 92.238 | 92.335 | 92.173 | 59.612 | 95.534 | 100 |  | 100 | 91.462 | 88.551 | 89.154 | 85.957 | 86.667 | 86.481 | 80.992 | 81.127 | 82.569 | 82.473 | 80.895 | 81.174 |
| RhQU44 | 84.787 | 84.819 | 84.755 | 84.496 | 92.518 | 92.238 | 92.335 | 92.173 | 59.612 | 95.534 | 100 | 100 |  | 91.462 | 88.551 | 89.154 | 85.957 | 86.667 | 86.481 | 80.992 | 81.127 | 82.569 | 82.473 | 80.895 | 81.174 |
| RhQU51 | 84.787 | 84.819 | 84.755 | 84.496 | 89.291 | 89.205 | 89.237 | 89.14 | 59.585 | 91.138 | 91.462 | 91.462 | 91.462 | **g** | 88.235 | 89.8 | 86.776 | 86.934 | 86.231 | 81.131 | 81.266 | 82.091 | 82.091 | 80.383 | 81.429 |
| RhOK10 | 84.409 | 84.442 | 84.377 | 84.119 | 87.417 | 87.33 | 87.33 | 87.298 | 60.388 | 88.551 | 88.551 | 88.551 | 88.551 | 88.235 | **f** | 86.645 | 83.826 | 84.994 | 84.939 | 80.686 | 80.57 | 81.561 | 81.529 | 80.332 | 81.302 |
| Rh_cya | 85.71 | 85.742 | 85.677 | 85.419 | 87.505 | 87.355 | 87.419 | 87.258 | 58.527 | 89.671 | 89.154 | 89.154 | 89.154 | 89.8 | 86.645 |  | 90.422 | 88.63 | 86.194 | 81.04 | 81.049 | 82.304 | 82.304 | 81.266 | 82.323 |
| Rh_sp1 | 83.281 | 83.312 | 83.249 | 82.997 | 84.854 | 84.833 | 84.928 | 84.802 | 56.207 | 86.083 | 85.957 | 85.957 | 85.957 | 86.776 | 83.826 | 90.422 |  | 85.444 | 82.557 | 78.39 | 78.545 | 79.925 | 79.925 | 78.389 | 79.826 |
| Rh_poly | 85.229 | 85.262 | 85.197 | 84.939 | 86.179 | 86.028 | 86.028 | 85.964 | 57.393 | 86.472 | 86.667 | 86.667 | 86.667 | 86.934 | 84.994 | 88.63 | 85.444 |  | 83.366 | 79.53 | 78.784 | 80.899 | 80.931 | 79.923 | 80.268 |
| RhOK5 | 82.537 | 82.569 | 82.505 | 82.247 | 85.283 | 85.133 | 85.197 | 85.068 | 57.578 | 86.611 | 86.481 | 86.481 | 86.481 | 86.231 | 84.939 | 86.194 | 82.557 | 83.366 | **h** | 79.148 | 79.62 | 80.72 | 80.88 | 79.808 | 79.464 |
| Rh_dob | 80.527 | 80.559 | 80.496 | 80.241 | 79.996 | 80.038 | 80.006 | 80.038 | 53.763 | 80.801 | 80.992 | 80.992 | 80.992 | 81.131 | 80.686 | 81.04 | 78.39 | 79.53 | 79.148 |  | 91.07 | 86.136 | 86.041 | 81.784 | 81.841 |
| Rh_nek | 79.399 | 79.43 | 79.367 | 79.051 | 79.882 | 79.861 | 79.829 | 79.861 | 53.768 | 81.127 | 81.127 | 81.127 | 81.127 | 81.266 | 80.57 | 81.049 | 78.545 | 78.784 | 79.62 | 91.07 |  | 86.134 | 86.039 | 80.214 | 80.979 |
| Rh_per | 81.592 | 81.624 | 81.561 | 81.306 | 81.156 | 81.071 | 81.103 | 81.071 | 54.933 | 82.122 | 82.569 | 82.569 | 82.569 | 82.091 | 81.561 | 82.304 | 79.925 | 80.899 | 80.72 | 86.136 | 86.134 |  | 99.712 | 80.925 | 82.606 |
| Rh_sp2 | 81.624 | 81.656 | 81.592 | 81.338 | 81.156 | 81.071 | 81.103 | 81.071 | 54.965 | 82.091 | 82.473 | 82.473 | 82.473 | 82.091 | 81.529 | 82.304 | 79.925 | 80.931 | 80.88 | 86.041 | 86.039 | 99.712 |  | 80.925 | 82.669 |
| BOU83331 | 80.467 | 80.499 | 80.435 | 80.243 | 79.587 | 79.821 | 79.757 | 79.757 | 54.068 | 80.895 | 80.895 | 80.895 | 80.895 | 80.383 | 80.332 | 81.266 | 78.389 | 79.923 | 79.808 | 81.784 | 80.214 | 80.925 | 80.925 |  | 79.264 |
| RhOK1 | 80.632 | 80.664 | 80.6 | 80.408 | 80.238 | 80.217 | 80.185 | 80.217 | 54.692 | 81.557 | 81.174 | 81.174 | 81.174 | 81.429 | 81.302 | 82.323 | 79.826 | 80.268 | 79.464 | 81.841 | 80.979 | 82.606 | 82.669 | 79.264 | **a** |

**Fig. S1.** Visualization of the barcode gap between interspecific (purple) and intraspecific genetic similarities (red) among the nine putative species of rhytidocystids infecting microturbellarians based on the data shown in Table S6.


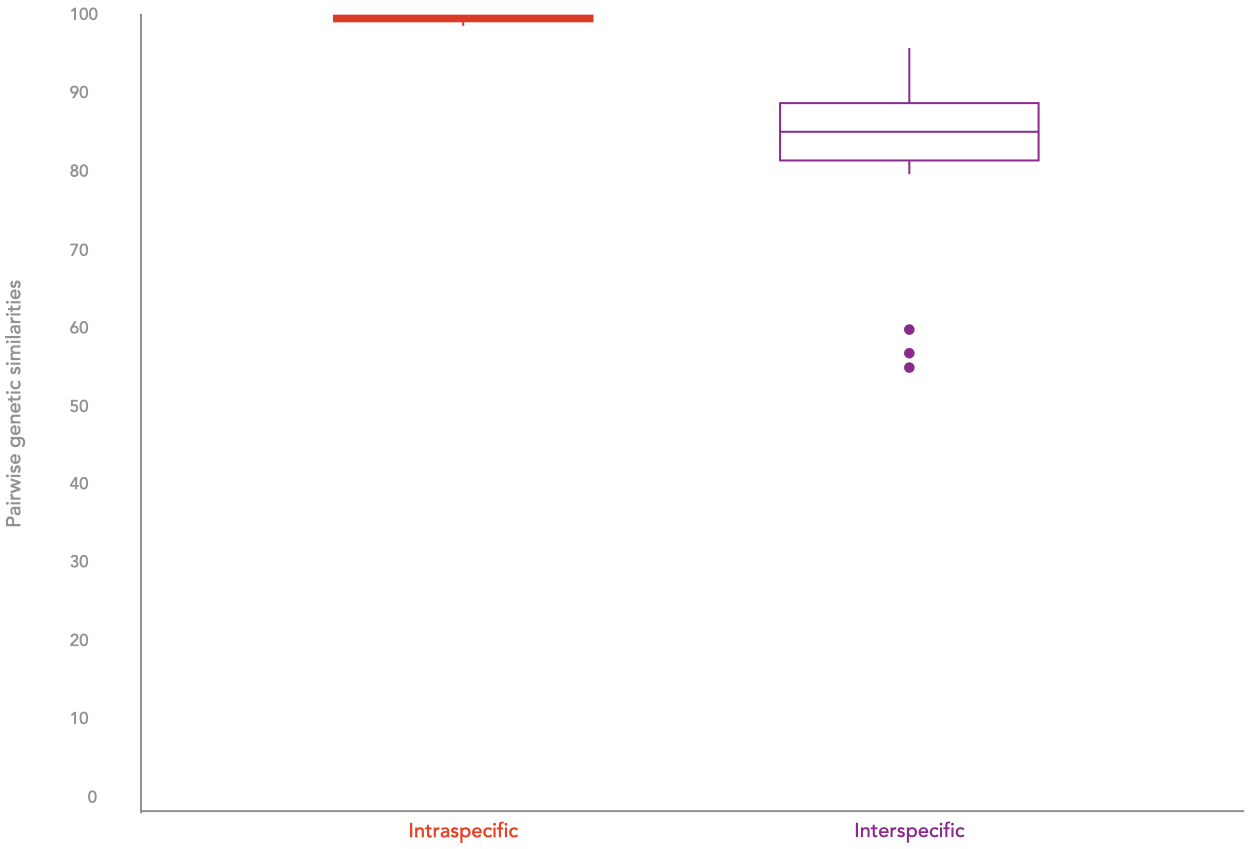

Supplement: Supplementary file 1 — Supplementary Information. [file 41598_2023_48233_MOESM1_ESM.docx]
